# Knowledge-enhanced biomedical named entity recognition and normalization: application to proteins and genes

**DOI:** 10.1186/s12859-020-3375-3

**Published:** 2020-01-30

**Authors:** Huiwei Zhou, Shixian Ning, Zhe Liu, Chengkun Lang, Zhuang Liu, Bizun Lei

**Affiliations:** School of Computer Science and Technology, Dalian University of Technology, Chuangxinyuan Building, No.2 Linggong Road, Ganjingzi District, Dalian, 116024 Liaoning China

**Keywords:** Entity recognition, Entity normalization, Knowledge base, Attention mechanism, Contextual word representations

## Abstract

**Background:**

Automated biomedical named entity recognition and normalization serves as the basis for many downstream applications in information management. However, this task is challenging due to name variations and entity ambiguity. A biomedical entity may have multiple variants and a variant could denote several different entity identifiers.

**Results:**

To remedy the above issues, we present a novel knowledge-enhanced system for protein/gene named entity recognition (PNER) and normalization (PNEN). On one hand, a large amount of entity name knowledge extracted from biomedical knowledge bases is used to recognize more entity variants. On the other hand, structural knowledge of entities is extracted and encoded as identifier (ID) embeddings, which are then used for better entity normalization. Moreover, deep contextualized word representations generated by pre-trained language models are also incorporated into our knowledge-enhanced system for modeling multi-sense information of entities. Experimental results on the BioCreative VI Bio-ID corpus show that our proposed knowledge-enhanced system achieves 0.871 *F*1-score for PNER and 0.445 *F*1-score for PNEN, respectively, leading to a new state-of-the-art performance.

**Conclusions:**

We propose a knowledge-enhanced system that combines both entity knowledge and deep contextualized word representations. Comparison results show that entity knowledge is beneficial to the PNER and PNEN task and can be well combined with contextualized information in our system for further improvement.

## Background

With the rapid development of computer technology and biotechnology, the number of biomedical literature is growing rapidly. These biomedical literatures contain a wealth of valuable knowledge, which can be used to promote biomedical development and help people improve their living environment. Furthermore, it is well recognized that the adoption of common database identifiers (IDs) could facilitate data integration and re-use. However, manually annotating them from massive biomedical literature is labor-intensive and costly. New methods and tools need to be developed to support more effective and consistent extraction of biomedical entities and their IDs, thereby facilitating downstream applications such as relation extraction [1] and knowledge base completion [2].

For this purpose, the BioCreative VI Track 1 proposed a challenging task (called Bio-ID Assignment), which focused on entity tagging and ID assignment [3]. There were two specific subtasks in Track 1: 1) biomedical named entity recognition (BioNER) and 2) normalization (BioNEN), also known as disambiguation. The first subtask aimed at automatically recognizing biomedical entities and their types from texts; and the second subtask was to associate entity mentions in texts with their corresponding common IDs in knowledge bases.

BioNER has been widely studied. Most existing approaches treat this problem as a sequence labeling task, which can be handled through traditional machine learning (ML)-based models (e.g., Hidden Markov Models and Conditional Random Fields) with complex feature engineering [4, 5]. Although effective, the design of features is labor-intensive and time-consuming. To overcome this drawback, neural networks were proposed to automatically extract features based on word embedding technology [6–8]. They constructed feature representations through multi-layer neural networks without relying on complicated feature engineering. Among them, bidirectional long short-term memory with conditional random field model (BiLSTM-CRF) exhibited promising results [6].

Compared with BioNER, BioNEN is a more challenging task. Previous work on this subtask was largely based on domain-specific dictionaries or heuristic rules [9, 10], and could achieve relatively high performance. However, these methods have a heavy reliance on the completeness of dictionaries and the design of rules. Therefore, it could be difficult to apply them to new datasets or shift them to new domains. Later, some work [11, 12] proposed to convert mentions and candidate entities into a common vector space, and then disambiguated candidate entities by a scoring function (e.g., cosine similarity). In recent years, neural network-based approaches have shown considerable success in entity normalization [13–15]. These methods used neural architectures to learn the context representations around an entity mention and calculated the context-entity similarity scores to determine which candidate is a correct assignment.

**Fig. 1:**
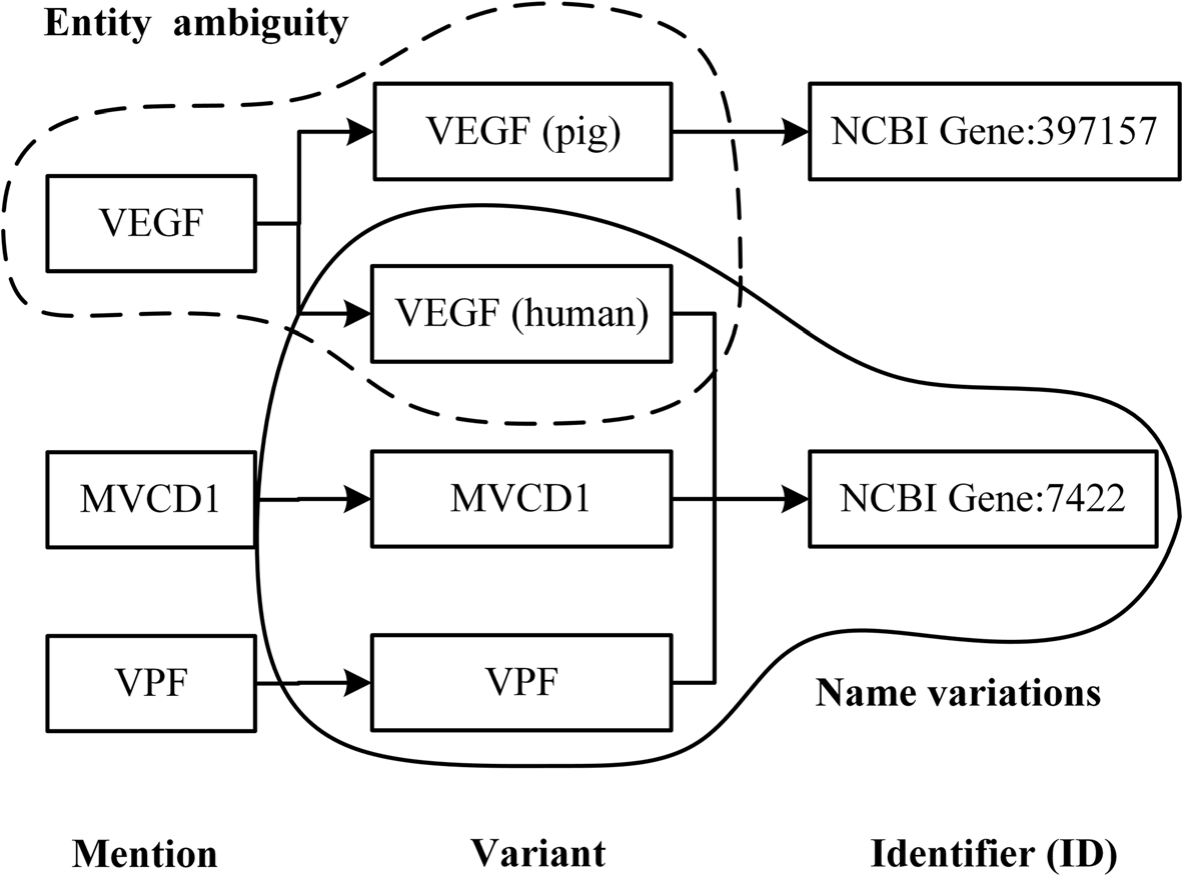
Illustration of structure information of entities. The first column represents the mention, the middle column represents the variant corresponding to the mention, and the last column represents the entity ID. The solid line box represents the name variations and the arrow in this side means that different variants can correspond to the same ID in the KB. The dashed box represents the entity ambiguity and the arrow in this side means that the same mention can have several variants with different ID in the KB

Although many studies have been made for the BioNER and BioNEN, yet challenges still exist. One is the **name variations**, which means that a named entity may have multiple surface forms, such as its full name, partial names, morphological variants, aliases and abbreviations [16]. The other is **entity ambiguity**, which means that an entity mention could possibly correspond to different entity IDs [16, 17]. Take Fig. 1 as an example to illustrate. In the solid line box of Fig. 1, the variants “VEGF (human)”, “MVCD1” and “VPF” all represent the same gene entity (vascular endothelial growth factor), whose ID is “NCBI Gene: 7422”. This is the name variations problem (synonym). The arrow in this side means that different variants can correspond to the same ID in the KB. In the dashed box, the variants “VEGF (human)” and “VEGF (pig)” have the same entity name, but correspond to different genus IDs (“NCBI Gene: 7422” and “NCBI Gene: 397157”, respectively). This is the entity ambiguity problem (polysemy). The arrow in this side means that the same mention can have several variants with different IDs in the KB.

Large-scale Biomedical Knowledge bases (KBs) such as UniProt [18] and NCBI Gene [19] contain rich information about the protein/gene entities and structural relationship between them. This information is quite useful for solving the above two issues. Luo et al. [20] and Akhondi et al. [21] showed that the information of prior chemical entity name provided by domain dictionaries could help boost the NER performance. However, the existing structural information of entities and how to use them for the Bio-ID Assignment task has not yet been well studied.

Besides, the multi-sense information of words has been leveraged and empirically verified to be powerful in many sequence labeling tasks [22, 23]. Peters et al. [22] proposed a deep contextualized word representation method, called Embeddings from Language Models (ELMo) [22]. This method directly adopted pre-trained bi-directional Language Models (biLMs) to integrate both semantic and multi-sense information of words as context-dependent embeddings. As far as we know, ELMo has not been well explored in biomedical domains.

In this paper, we propose a novel knowledge-enhanced system that could employ rich entity knowledge and deep contextual word representations for protein/gene named entity recognition (PNER) and normalization (PNEN). Specifically, entity name knowledge in KBs is introduced into a BiLSTM-CRF model to recall more protein/gene mentions. Then, structural knowledge of entities is encoded by an autoencoder into ID embeddings, which are used for entity disambiguation during the PNEN phase. To further explore the **entity ambiguity** issue, ELMo is also incorporated into our system to capture underlying meanings for each word. Experiments on the BioCreative VI Bio-ID corpus show that our proposed knowledge-enhanced system could effectively leverage prior knowledge and achieves state-of-the-art performance on both PNER and PNEN subtasks.

The contributions of this work are summarized as follows:

- We explore the effect of ELMo representations on entity recognition and normalization in biomedical domains. Experimental results show that it could accurately capture context-dependent aspects of word meaning, therefore effectively improving the performance of PNER and PNEN.
- We integrate structural knowledge of entities into ID embeddings, which can be beneficial to remedy the **entity ambiguity** issue faced by PNEN.
- Entity name knowledge, which can be used as prior clues to better address the issue of **name variations**, is also incorporated into our system.

## Results

### Experiment setup

#### Dataset

Our experiments are conducted on the corpus published by BioCreative VI Bio-ID Track1 [3], which is drawn from annotated figure panel captions from SourceData [24] and is converted into BioC format along with the corresponding full text articles.

Bio-ID corpus contains a training set and a test set. The training set consists of 13,573 annotated figure panel captions corresponding to 3658 figures from 570 full length articles, with a total of 51,977 annotated Protein/Gene IDs. The test set consists of 4310 annotated figure panel captions from 1154 figures taken from 196 full length articles, with a total of 14,232 annotated Protein/Gene IDs.

Table 1 shows the statistical results of the number of IDs that an entity mention has (entity ambiguity) on the BioID corpus. For each target mention in the BioID corpus, we estimate ambiguity as the number of different IDs associated to it by human annotators. From Table 1, we can know that: (1) many mentions tend to be highly skewed, in the sense that they usually refer to one specific entity; (2) nearly one-third of mentions correspond to two or more different IDs; (3) the ambiguity rate per ambiguous mention is 2.79 on the training set and 2.41 on the test set.

**Table 1:** Statistics of entity ambiguity for the Bio-ID corpus

Properties	Training set	Test set
# Mentions	4440	1715
# Monosemous	3031	1265
# Polysemous/Ambiguity Rate	1409 / 2.79	450 / 2.41

The left column reports four types of attributes, which are the number of unique proteins/genes mention terms (#Mentions), the number of #Mentions with only one entity ID attested in the corpus (#Monosemous), the number of #Mentions with two or more IDs attested in the corpus (#Polysemous), and the average number of candidate IDs that a *polysemous* target mention has (Ambiguity Rate)

Table 2 shows the statistical results of the number of entity variants corresponding to a specific entity ID (**name variations**) on the BioID corpus. We compute the synonymy rate as the number of different variants that can be used to name a particular ID. From Table 2, we can see that: (1) for the 5282 IDs present in the Training set and 1980 IDs present in the Test set, most entities are associated with only a single variant (78% in the Training set and 85% in the Test set respectively); (2) and the synonymy rate is lower, 2.46 on the training set and 2.26 on the test set.

**Table 2:** Statistics of name variations for the Bio-ID corpus

Properties	Training set	Test set
# IDs	5282	1980
# Single Var.	4133	1689
# Multiple Var. / Synonymy Rate	1149 / 2.46	291 / 2.26

The left column tabulates four types of attributes, which are the number of unique entity IDs (#IDs), the number of #IDs with only one variant (#Single Var.), the number of #IDs with two or more variants (#Multiple Var.), and the average number of variants that a *multiple var.* target ID has (Synonymy Rate)

#### Negative sampling

Since our disambiguation model is only given training samples for correct ID assignments, negative sampling is needed to automatically generate samples of corrupt assignments. For each context-ID pair (*C*, *s*), where *s* is the correct ID assignment for the context *C* around the entity mention, we produce some negative samples with the same context *C* but with a different entity ID *s*^′^. Following Eshel et al. [13], we uniformly sample out of the candidate IDs of each mention to obtain a corrupt *s*^′^ for forming each negative sample (*C*, *s*^′^).

#### Training details

Throughout our experiments, a word is initialized with 200-dimensional pre-trained word embeddings [25], which are trained on the openly available biomedical literature (∼5B words) using the word2vec tool. The dimensions for character, part-of-speech (POS), chunking, and knowledge features (KFs) are 50, 25, 10, and 15, respectively. Deep contextualized word representations ELMo is 1024-dimensional generated by biLMs pre-trained on a corpus with approximately 30 million sentences [22]. For both PNER and PNEN, we fine-tune all the parameters during training to improve the performance.

UniProt [18] and the NCBI Gene [19] KBs are used for entity knowledge extraction as well as candidate ID generation. The versions of UniProt and NCBI Gene used in our experiments are 2018_11 and 04-Dec-2018, respectively. The disambiguation model is trained with fixed-size left and right contexts (*n*_2_ = 10 words in each side excluding stop words and punctuation). Mini-batch size is set to 8 for both models. We fixed the dropout rate at 0.5 during training to ease the overfitting problem.

In the following experiments, we randomly chose 80% of the training set to be the actual training set and the remaining 20% to be the validation set. The training set is used to fit the parameters of the model, the validation set is used to evaluate the performance of our models and choose the hyper-parameters settings associated with the best performance (hyper-parameter tuning), the test set is used to assess the performance of the final chosen model by the official evaluation scripts provided by the BioID shared task.

The PNER task is evaluated on strict entity span matching, i.e. the character offsets have to be identical with the gold standard annotations. For the PNEN task, only the normalized IDs returned by the systems are evaluated. The performance of the systems is reported as precision (*P*), recall (*R*) and *F*1-score (*F*1) on corpus level.

### Performance

#### PNER performance

In this experiment, we explore the effects of different features representations on the performance of our recognition model. Table 3 shows the results of different combinations of these features on the test set. We first take the BiLSTM-CRF model with word embedding and character embedding $\left[{x}_t^w,{x}_t^c\right]$ as the **baseline** for comparison.

**Table 3:** Results with KF/ELMo representations on the test set for PNER

Model	Strict Match			Overlap Match		
	*P*	*R*	*F*1	*P*	*R*	*F*1
Baseline	0.749	0.816	0.781	0.800	0.871	0.834
+ linguistic features	0.817	0.764	0.789	0.868	0.812	0.839
+ linguistic features + KFs	0.776	**0.845**	0.809	0.820	**0.893**	0.855
+ linguistic features + ELMo	**0.826**	0.801	0.813	**0.874**	0.847	0.860
+ linguistic features + KFs + ELMo (ours)	0.815	0.812	**0.814**	0.873	0.869	**0.871**

**Strict match** criteria require that the predicted entity and the gold standard annotations have to match exactly at the byte offset; and **overlap match** criteria allows a match if the predicted entity overlaps with the gold annotation at all. The highest scores are highlighted in bold. We tune the hyper-parameters through the validation set and use the official evaluation script to assess the performance of the final chosen model on the test set

From Table 3, we can see that the addition of linguistic features (POS tagging and chunking) contributes to the PNER task on both the strict and overlap criteria, but only achieves a small improvement of 0.8 and 0.5% in *F*1-score, respectively. On the basis of linguistic features, the addition of KFs and ELMo representations bring a significant improvement in the PNER performance.

Take a look at the **overlap match** criteria, the addition of KFs increases the *F*1-score from 0.839 to 0.855 (1.6% improvement), especially showing substantial recall gains when comparing with others. This demonstrates that the rich information of prior protein/gene entities provided by KFs helps recognize more entity variants and proves the validity of entity name knowledge on the **name variations** issue.

Similarly, the addition of ELMo representations increases the *F*1-score by 2.1% from 0.839 to 0.860. Although the recall gains brought by the ELMo is not as good as that of KFs, it is capable of modeling the multi-sense information of words across vary linguistic context, resulting in an increase in precision. In other words, this allows the BiLSTM-CRF model to better understand the context information to accurately distinguish entities and non-entities. Moreover, ELMo can complement the context-free nature of traditional word embedding to represent context-dependent information.

When all additional features (linguistic features, KFs and ELMo representations) are added, the best performance (0.814 *F*1-score at the strict criteria and 0.871 *F*1-score at the overlap criteria) is achieved. This proves that there exists a complementary relationship between KFs and ELMo, thus they can balance the recall and precision of the BiLSTM-CRF model on the PNER subtask.

#### PNEN performance

In this experiment, we explore the effects of the architecture of our disambiguation model on the test set based on the above PNER model selected by the validation set. Since the ELMo representation and the gating mechanism have been proven to be effective, they will be added directly to the entity disambiguation model without being explored. Five common sequence encoders are used for context representation learning, which are shown below.

##### LSTM and GRU

Firstly, we explore the standard recurrent encoders with either Long Short-Term Memory (LSTM) or Gated Recurrent Units (GRU) for sequence encoding. The last hidden state is used to represent a sequence.

##### BiLSTM and BiGRU

To preserve information from both past and future, we also consider bidirectional LSTM/GRU that concatenates the last hidden state of the forward direction, and the last hidden state of the backward direction to represent a sequence.

##### Hierarchical-ConvNet

Inspired by Zhao et al. [26], we introduce a hierarchical convolutional network which concatenates different representations of the sequence at four different levels of convolutional layers. In each layer, a representation *u*_*i*_ is computed by a max-pooling operation over the feature maps. The final sequence representation ${h}_t^{se}=\left[{u}_1,{u}_2,{u}_3,{u}_4\right]$ is the concatenation of *u*_*i*_ from each layer.

In addition, two kinds of attention mechanism are proposed to verify the effect of ID embedding. The first is **Knowledge-based attention**, which is designed to focus on contextual words that are more relevant to candidate IDs (mentioned in the section *2.3.3 Entity Disambiguation*). The second is **Self-attention** [27], which is similar to the former except that pre-trained ID embeddings is not considered when calculating the attention score, as shown below:

1 $$ {e}_t=\tanh \left({\mathbf{W}}_a^L{h}_t^{se}+{b}_a^L\right). $$

Table 4 shows the results of different model architectures on the test set. From Table 4, we can see that:

1. The recurrent encoders (**GRU**, **LSTM**, **BiLSTM** and **BiGRU**) achieve better performance than **Hierarchical-ConvNet**. Recurrent encoders are suitable to capture the long-term dependencies within sequences, while **Hierarchical-ConvNet** is suitable to capture the local features. In most cases, entity disambiguation relies predominantly on global features rather than local features.
2. Both **BiLSTM** and **BiGRU** perform well and are superior to unidirectional models (**LSTM** and **GRU**). Compared with the unidirectional models, the bidirectional models could capture context information more comprehensively.
3. By incorporating attention mechanism, the above five sequence encoders have achieved performance improvements, regardless of the introduction of **Knowledge-based attention** or **Self-attention**. The possible reason is that the attention mechanism could flexibly capture global and local connections and better model long-term dependencies to capture important context information between elements in a sequence.
4. **Knowledge-based attention** could effectively fuse knowledge and context representations and outperform the **Self-attention** mechanism. With the help of ID embeddings learned by autoencoder, **Knowledge-based attention** brings more benefits to PNEN and significantly increases its *F*1-score under both micro- and macro-averages. We attribute it to the following two aspects. On one hand, **Knowledge-based attention** mechanism could find important contexts related to candidate ID. On the other hand, the knowledge representations obtained from KBs through ID representation learning could provide valid information for PNEN. Knowledge representations could efficiently encode prior entity structural knowledge in a low-dimensional space and significantly improve the performance of PNEN.
5. Compared to **BiLSTM + Knowledge-based attention**, **BiGRU + Knowledge-based attention** wins with a slight advantage and achieves the highest Micro-averaged *F*1-score of 0.445 in all models, which means that it could better integrate context information and prior knowledge through the proposed attention mechanism.

**Table 4:** Results with different choices of model architecture on the test set for PNEN

Model	Micro-averaged			Macro-averaged		
	*P*	*R*	*F*1	*P*	*R*	*F*1
LSTM	0.486	0.388	0.431	0.537	0.443	0.395
+ Self-attention	0.490	0.391	0.435	0.550	0.452	0.405
+ Knowledge-based attention	0.495	0.395	0.440	0.559	**0.465**	**0.417**
GRU	0.484	0.387	0.430	0.548	0.454	0.406
+ Self-attention	0.491	0.393	0.436	0.558	0.461	0.414
+ Knowledge-based attention	0.495	0.395	0.439	0.551	0.454	0.407
BiLSTM	0.486	0.389	0.432	0.541	0.446	0.398
+ Self-attention	0.493	0.394	0.438	0.552	0.456	0.408
+ Knowledge-based attention	0.499	0.400	0.444	0.559	0.461	0.414
BiGRU	0.487	0.389	0.433	0.542	0.446	0.398
+ Self-attention	0.497	0.397	0.441	0.558	0.462	0.415
+ Knowledge-based attention	**0.501**	**0.400**	**0.445**	**0.562**	0.464	0.416
Hierarchical-ConvNet	0.468	0.375	0.416	0.522	0.429	0.381
+ Self-attention	0.473	0.378	0.420	0.529	0.434	0.386
+ Knowledge-based attention	0.483	0.386	0.429	0.532	0.441	0.392

Only the normalized IDs returned by the systems are evaluated on both **micro-averaged** and **macro-averaged** metrics. **Micro-averaged** calculates metrics globally by counting the total true positives, false negatives and false positives, **macro-averaged** calculates metrics for each label in documents and finds their unweighted mean. The highest scores are highlighted in bold. We tune the hyper-parameters through the validation set and use the official evaluation script to assess the performance of the final chosen model on the test set.

## Discussion

### Comparison with related work for PNER

We compared our approach with other related work on the PNER subtask and the results are shown in Table 5.

**Table 5:** Comparison with related work on the PNER subtask

System	Strict Match			Overlap Match		
	*P*	*R*	*F*1	*P*	*R*	*F*1
Sheng et al. [28]	0.509	0.613	0.556	0.686	0.826	0.749
Kaewphan et al. [29]	0.729	0.739	0.734	0.825	0.836	0.831
Kaewphan et al. [30]	0.764	0.768	0.766	–	–	–
Ours	**0.815**	**0.812**	**0.814**	0.873	0.869	0.871

**Strict match** criteria require that the predicted entity and the gold standard annotations have to match exactly at the byte offset; and **overlap match** criteria allows a match if the predicted entity overlaps with the gold annotation at all. The highest scores are highlighted in bold.

Kaewphan et al. [29] used a publicly available NER toolkit NERsuite with one-hot represented word and POS tagging as input for PNER. Additional dictionary features were also used for their experiments, but there was no clear performance improvement in either strict or overlap criteria. Their recognition approach achieved the highest rank on the PNER subtask (0.734 and 0.831 F1-scores under both matching criteria). However, traditional ML-based methods need extensive feature engineering, which is time-consuming and labor intensive. Based on their previous work [29], Kaewphan et al. [30] further developed a BiLSTM-CRF based model, which used character embeddings learned by a Convolutional Neural Network (CNN) and the predictions from their original NERsuite model [29] as inputs of the recognition model. Neural network-based methods bring significant improvement in PNER performance (3.2% F1-score improvement under strict criteria than before). However, they did not use entity name knowledge or ELMo representations, resulting in a 4.8% F1-score lower than our method. Sheng et al. [28] also constructed a BiLSTM-CRF model that used only word and character as inputs, without relying on the help of any other external features.

Comparing with these approaches, our model incorporates multi-sense information of words and entity name knowledge in KBs. Therefore, our model gets relatively balanced precision and recall while both are improved, which outperforms approaches mentioned above.

### Comparison with related work for PNEN

Similarly, we compared our work with other related work on the PNEN subtask. The results are shown in Table 6.

**Table 6:** Comparison with related work on the PNEN subtask

System	Micro-averaged		
	*P*	*R*	*F*1
Sheng et al. [28]	0.170	0.224	0.193
Kaewphan et al. [29]	0.472	0.343	0.397
Kaewphan et al. [30]	0.445	0.388	0.415
Ours	**0.501**	**0.400**	**0.445**

The highest scores are highlighted in bold

Kaewphan et al. [29] applied exact string matching to retrieve candidate IDs of protein/gene mentions based on KBs. For the ambiguous mentions with multiple candidate IDs, some heuristic rules were developed for disambiguating protein/gene mentions and uniquely assigning an ID. Their normalization approach achieved the highest rank on this PNEN subtask (0.397 micro-averaged *F*1-score). Typically, hand-crafted rules are clear and effective, but they are inflexible and hard to expand to a new dataset. Kaewphan et al. [30] used the same method as their previous work [29] to perform PNEN, but based on their new recognition method. Compared with their previous results, they achieved 1.8% micro-averaged *F*1-score improvement from 0.397 to 0.415. This shows that their normalization approach depends on the performance of PNER to a large extent.

Sheng et al. [28] compiled a contextual dictionary based on the training set and then checked if the entity mention was in this contextual dictionary. If so, they normalized the mention to the known ID that shared the most contextual words with the sequence the entity belonged to. For cases without matched IDs in the compiled dictionary, they used the same UniProt API and NCBI Gene API as ours, to search for candidate IDs and directly assigned the first ID match to ambiguous mentions. Since no disambiguation models were used to pick candidate IDs, their approach achieved a relatively low precision and recall on the PNEN subtask.

Our normalization approach outperforms the above related approaches and achieves a state-of-the-art result (0.445 micro-averaged *F*1-score). We attribute this to the validity of our disambiguation model, which accurately models the context representation through rich structural knowledge of entities in KBs.

### Influences of the training data size

We further explored the influences of the training data size. We first divided the Bio-ID training data into eight parts and then added each part to training set one by one. In this case, we trained eight models with different sizes of training sets.

**Fig. 2:**
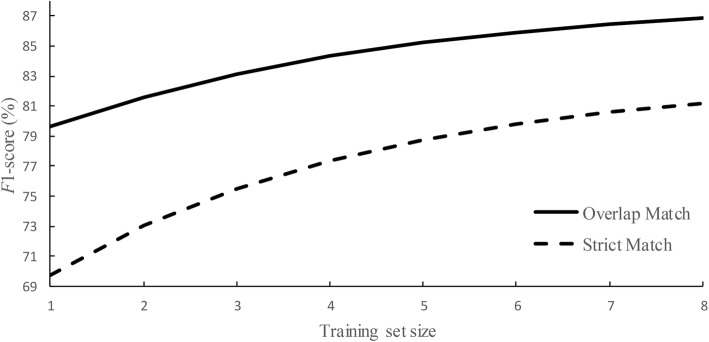
Training data size vs. PNER *F*1-score

**Fig. 3:**
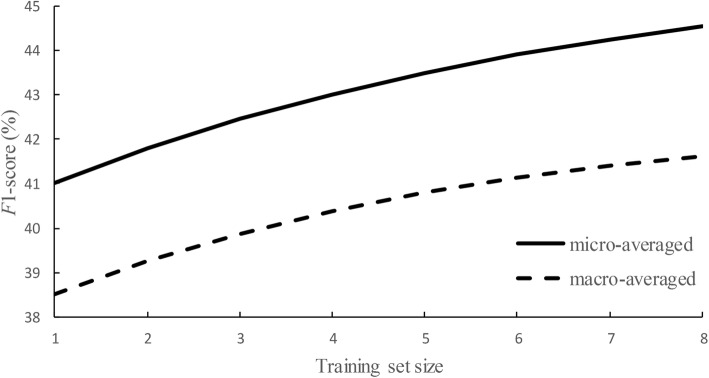
Training data size vs. PNEN *F*1-score

Figure 2 shows the trend of PNER results with the size of training set increasing. From Fig. 2 we can see that the *F*1-scores of the PNER subtask increase gradually when the training set size increases. To estimate the asymptotic *F*1-score for PNER, we defined a non-linear function *F*1_PNER_ = *i* + *jm*^*n*^ to fit the results of Strict Match and Overlap Match criterias as follows:

2 $$ F{1}_{\mathrm{PNER}}^{\mathrm{strict}}=0.82948-0.17636{(0.750)}^n $$

3 $$ F{1}_{\mathrm{PNER}}^{\mathrm{overlap}}=0.88179-0.11142{(0.767)}^n $$

where *n* is the number of training set part. These functions illustrate that as the training set increases, the asymptotic *F*1-scores under strict and overlap criteria could reach to about 0.829 and 0.882, respectively. Similarly, Fig. 3 shows the same information for PNEN subtask and the following estimations are obtained:

4 $$ F{1}_{\mathrm{PNEN}}^{\mathrm{micro}}=0.46260-0.06155{(0.852)}^n $$

5 $$ F{1}_{\mathrm{PNEN}}^{\mathrm{macro}}=0.42573-0.05011{(0.813)}^n $$

These functions show similar results. As the training dataset increases, the asymptotic *F*1-scores under micro- and macro-averages could reach to about 0.463 and 0.426, respectively.

### Error analysis

We analyzed the incorrect output of our knowledge-enhanced system on the PNEN subtask, and divided them into the following four types:

1. PNEN FPs caused by PNER FPs: false positives in PNER (PNER FPs) could cause PNEN FPs, which refers to some non-entity words are incorrectly recognized as entities during the PNER phase, resulting in normalization errors during the PNEN phase.
2. PNEN FNs caused by PNER FNs: false negatives in PNER (PNER FNs) could cause PNEN FNs, which refers to that some entities are not recognized during the PNER phase and they cannot be normalized.
3. PNEN FPs caused by Missed ID: true positives in PNER but false positives in PNEN (PNEN FPs), which is the case that correct ID of the ambiguous mention is not included in the result retrieved by candidate ID generation.
4. PNEN FPs caused by Incorrect ID: true positives in PNER but false positives in PNEN (PNEN FPs), which is the case that our system fails to assign the correct ID to the ambiguous mention.

See Table 7 for more details. PNER FPs propagate to the PNEN phase and cause 995 normalization errors, with a proportion of 17.59%. PNER FPs is that some non-entity words are incorrectly recognized as entities. Take the sentence (1) predicted by our knowledge-enhanced system as an example. The word “*Miro*” denoted by wave line looks much like the annotated entity “*Miro1*” according to its context, but is noise actually. Although we introduced deep contextual word representations ELMo into the system to capture details of the context, there are still errors in the PNER phase.

**Table 7:** Error analysis on the test set

PNER category	Error type	Definition	#Errors	Error Percentage
PNER FPs	PNEN FPs caused by PNER FPs	Non-entities incorrectly recognized as entities during the PNER phase	995	17.59%
PNER FNs	PNEN FNs caused by PNER FNs	Entities not recognized during the PNER phase	2561	45.27%
PNER TPs	PNEN FPs caused by Missed ID	Entities that do not include the correct ID in the result retrieved by candidate ID generation	1376	24.32%
	PNEN FPs caused by Incorrect ID	Entities assigned with the error ID by entity disambiguation	725	12.82%

Sentence (1): SyGCaMP5 and MtDsRed or *myc* [*protein:myc*] - ΔEF - *Miro1* [*NCBI* Gene*:8850*] - IRES - MtDsRed (ΔEF *Miro* [*NCBI* Gene*:8850*]) with and without *TTX* treatments. (predicted by our system).

PNER FNs propagate to the PNEN phase and cause 2561 normalization errors, with a proportion of 45.21%. Such errors are generally related to the domain-specific abbreviations. In sentence (1), the word “*TTX*” denoted by underline is a gene entity but not recognized by our system. We analyzed two causes that lead to many entities not being able to be recalled. One is that although a large amount of variant information exists in the introduced entity name knowledge, there is still no guarantee that the coverage of a large number of abbreviated variants is complete. The other is that the textual context around the entity mention is too general, which makes our system difficult to capture discriminative information to disambiguate mentions.

Though some ambiguous entity mentions are correctly recognized in the PNER phase, they are assigned incorrect ID in the PNEN phase. Such kind of errors can be further divided into two sub-categories, PNEN FPs caused by Missed ID and PNEN FPs caused by Incorrect ID. Take sentence (1) to help understand, the true positive “*Miro1*” should correspond to the ID “*NCBI* Gene*:59040*”, but an incorrect “*NCBI* Gene*:8850*” is assigned.

PNEN FPs caused by Missed ID is the case that correct ID of the ambiguous mention is not included in the result retrieved by candidate ID generation. Missed ID is usually related to the **entity ambiguity** issue. The more variants an ambiguous mention corresponds to, the more candidate IDs it may have, which makes the resulting candidate IDs (up to 5) more difficult to cover the correct ID during the PNEN phase. The missed ID sub-category brings 1376 errors, with a proportion of 24.35%.

PNEN FPs caused by Incorrect ID is the case that our system fails to assign the correct ID to the ambiguous mention. Although we add pre-trained ID embeddings to help context representation learning as accurately as possible, there are still 725 errors (12.83%) caused by incorrect classification.

## Conclusions

In this paper, we present a knowledge-enhanced system for biomedical named entity recognition and normalization with proteins and genes as the application target. For the **name variations** challenge, entity name knowledge is used for PNER to increase its recall rate. ELMo representations are also added to the recognition model for the purpose of improving the model precision. For the **entity ambiguity** challenge, we use an autoencoder to encode structural knowledge of entities into ID embeddings for better entity disambiguation. Experimental results on the BioCreative VI Bio-ID dataset verify that the proposed system outperforms the existing state-of-the-art systems on both PNER and PNEN subtasks, with the aid of these two kinds of knowledge and ELMo representations.

Our system implemented this task in two separate steps in a pipeline, which may lead to error propagation from PNER to PNEN as can be seen from the error analysis. As future work, we would like to construct a joint model that recognizes and normalizes protein/gene entities simultaneously, to reduce such error propagation by enabling feedback from PNEN phase to PNER phase. And, it allows entity recognition and normalization to interact with each other to jointly optimize PNER and PNEN.

## Methods

**Fig. 4:**
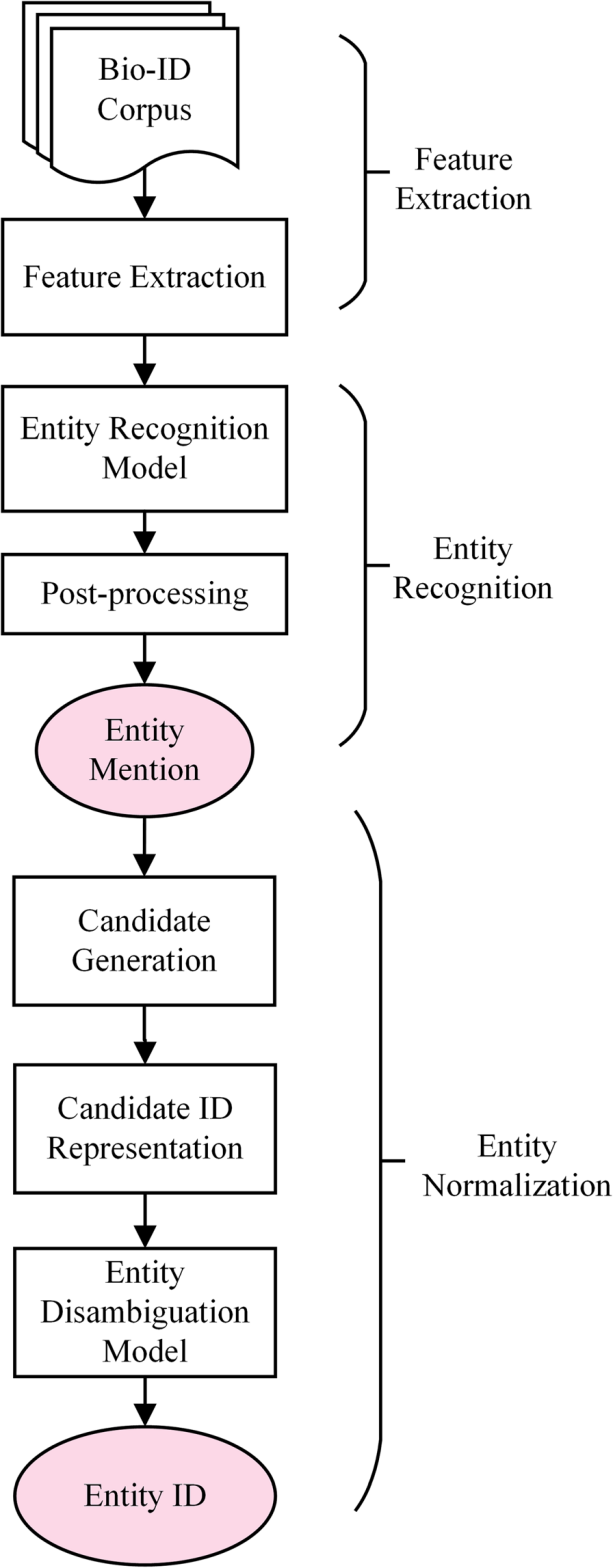
The workflow of our knowledge-enhanced system. The arrow means the workflow of the system, the rectangle indicates a specific operation or process, and the pink oval box indicates the results of entity recognition and normalization

In this section, we describe our knowledge-enhanced system for the Bio-ID Assignment task. Figure 4 shows the workflow of our system. It can be divided into three modules:

1. Feature extraction is performed on the original corpus, and six types of features are obtained and used as input to the entity recognition model.
2. Entity recognition is used to get the entity mentions. The extracted features are mapped to vector representations and concatenated together as inputs to the entity recognition model for entity mentions. To further improve the model performance, some heuristic rules are used to correct the predicted results output by the entity recognition model.
3. Entity normalization is used to generate candidate IDs and eliminate ambiguity for mentions. This module first generates candidate IDs for the mentions, which are then mapped to pre-trained ID embeddings. The candidate ID embeddings will then be fed to the entity disambiguation model along with the local contexts of the mention, for the purpose of picking the most likely one as the assignment result for the mention.

### Feature extraction

Refer to the practice of Tsai et al. [31], we employ the GENIA Tagger [32] to process input documents, including tokenization, POS tagging and chunking. All of these provide features for our BiLSTM-CRF model to further enrich the information of each word. In particular, to recognize more entity variants and alleviate the **name variations** issue, entity name knowledge contained in UniProt and NCBI Gene KBs is used to generate our KFs. Specifically, the longest possible match between the input word sequences and variant term of entities contained in KBs are first captured. Then, for each word in the match, it is encoded in BIO (Begin, Inside, Outside) tagging scheme to form KFs. Intuitively, KFs can augment the hint information for each entity mention by a large number of variant terms in KBs.

In addition, ELMo representation learned by a pre-trained LSTM-based multi-layer biLM is also extracted. The biLM takes character sequence of each word as input and encodes them with CNN and highway networks, whose output is then given to a two-layers BiLSTM with residual connections. Then, the combination of hidden states of each layer is performed to assign each word an ELMo representation. The following formula gives the ELMo representation for the *t*-th word,

6 $$ {x}_t^{elmo}=\gamma \sum \limits_{j=0}^L{l}_j{h}_{t,j}^{LM}. $$

where ${h}_{t,j}^{LM}$ is the *t*-th hidden state of *j*-th LSTM layer in the biLM, *l*_*j*_ is the softmax-normalized weight of *j*-th LSTM layer in the biLM. *γ* is the scalar parameter that allows the model to scale the entire ELMo representations.

ELMo can model multi-sense information of each word across different linguistic contexts and make the embeddings contain more contextual information.

### Entity recognition

**Fig. 5:**
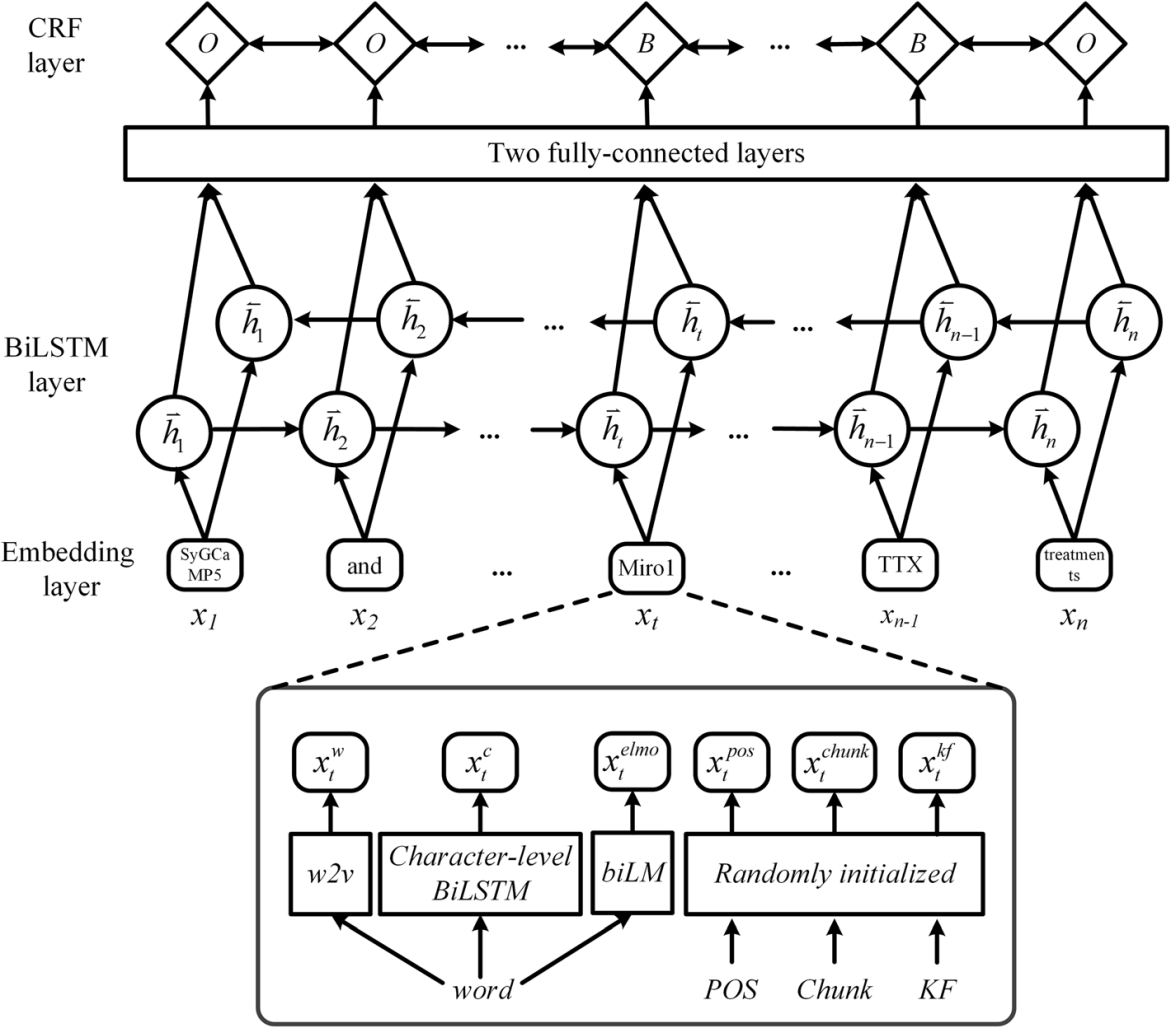
The architecture of BiLSTM-CRF model for PNER. In embedding layer, “w2v” means the word embeddings pre-trained using the word2vec tool, character embedding can be learned by Character-level BiLSTM, “biLM” means the pre-trained bi-directional Language Model ELMo, “Randomly initialized” means obtaining a corresponding vector in a random manner. Six feature representations of each word are concatenated together to form an input and fed to a BiLSTM layer. The last is the CRF layer, which is used to decode the best tag path in all possible tag paths. The input sentence is “SyGCaMP5 and MtDsRed or **myc** - ΔEF - **Miro1** - IRES - MtDsRed ( ΔEF Miro) with and without **TTX** treatments”. The output tag sequence is “OOOOBOOOBOOOOOOOOOOOBO”

The architecture of BiLSTM-CRF model for entity recognition is illustrated in Fig. 5. It mainly consists of three parts: Embedding layer, BiLSTM layer and CRF layer.

#### Embedding layer

Given an input feature sequence **W** = {*w*_1_, ..., *w*_*t*_, ..., *w*_*n*_} ∈ *ℝ*^*n*^, it is mapped to a feature vector sequence **X** = {*x*_1_, ..., *x*_*t*_, ..., *x*_*n*_} ∈ *ℝ*^*d* × *n*^ through the embedding layer, where *d* is the embedding dimension and *n* is the sequence length.

Each of the feature vectors *x*_*t*_ ∈ *ℝ*^*d*^ consists of the following six parts. A word embedding ${x}_t^w$ mapped by the word embedding matrix pre-trained by the word2vec tool, a character embedding ${x}_t^c$ learned from the character sequence of the word by character-level BiLSTM, an ELMo representation ${x}_t^{elmo}$ learned by the pre-trained LSTM-based multi-layer biLM, and the POS, chunking, knowledge feature vectors ${x}_t^{pos}$, ${x}_t^{chunk}$, ${x}_t^{kf}$ obtained by random initialization mapping.

The concatenation of the above six feature vectors yields the output of the embedding layer, which can be represented as follows:

7 $$ {x}_t=\left[{x}_t^w;{x}_t^c;{x}_t^{pos};{x}_t^{chunk};{x}_t^{kf};{x}_t^{elmo}\right]. $$

After that**, the feature vector sequence X will be taken as input to the next BiLSTM layer for context representation learning.**

#### BiLSTM layer

Long short-term memory (LSTM) is a specific type of recurrent neural network that models dependencies between elements in a sequence through recurrent connections. Here, we use one forward LSTM to compute a hidden state ${\overrightarrow{h}}_t= LSTM\left({x}_t,{\overrightarrow{h}}_{t-1}\right)\in {\mathbb{R}}^{d_2}$ of the sequence **X** from left to right at the *t*-th time step, and the other backward LSTM to compute a hidden state ${\overleftarrow{h}}_t= LSTM\left({x}_t,{\overleftarrow{h}}_{t+1}\right)\in {\mathbb{R}}^{d_2}$ of the same sequence in reverse, where *d*_2_ is the dimension of the hidden state. Then, the two hidden states are concatenated to form the final output ${h}_t=\left[{\overrightarrow{h}}_t;{\overleftarrow{h}}_t\right]$ of the BiLSTM layer at the *t*-th time step.

After that, the output of BiLSTM $\mathbf{h}=\left\{{h}_1,...,{h}_t,...,{h}_n\right\}\in {\mathbb{R}}^{2{d}_2\times n}$ is fed to a two-layer fully-connected neural network (FC) with tanh activation to predict the confidence score for each possible label of the word, which can be written as follow:

8 $$ \mathbf{P}=\mathbf{V}\left(\tanh \left(\mathbf{Wh}+\mathbf{b}\right)\right). $$

where $\mathbf{W}\in {\mathbb{R}}^{d_2\times 2{d}_2}$, $\mathbf{V}\in {\mathbb{R}}^{k\times {d}_2}$ and $\mathbf{b}\in {\mathbb{R}}^{d_2\times n}$ are the parameters that need to be trained, *k* is the number of distinct labels.

#### CRF layer

To model the dependencies across output tags, a Linear-Chain CRF layer is added on top of the BiLSTM layer to decode the best tag path in all possible tag paths. For the input sequence **X**, we consider **P** ∈ *ℝ*^*n* × *k*^ to be the matrix of scores output by the BiLSTM layer. The element *P*_*i*, *j*_ of the matrix corresponds to the score of the *j*-th tag of the *i*-th word. For a sequence of predictions **y** = {*y*_1_, ..., *y*_*t*_, ..., *y*_*n*_}, we define its score to be:

9 $$ score\left(\mathbf{X},\mathbf{y}\right)={\sum}_{i=1}^n\left({T}_{y_{i-1},{y}_i}+{P}_{i,{y}_i}\right). $$

where **T** is a matrix of transition scores such that *T*_*i*, *j*_ represents the score of a transition from the tag *i* to tag *j*. *y*_0_ and *y*_*n*_ are the extra start and end tags of a sequence and **T** is therefore a square matrix of size *k* + 2.

Then, a softmax is used to yield a probability of the path **y** by normalizing the above score over all possible tag path **y**^′^:

10 $$ P\left(\mathbf{y}|\mathbf{X}\right)=\frac{\exp \left( score\left(\mathbf{X},\mathbf{y}\right)\right)}{\sum_{{\mathbf{y}}^{\prime }}\exp \left( score\left(\mathbf{X},{\mathbf{y}}^{\prime}\right)\right)}. $$

During training, we maximize the log-likelihood of the correct tag sequence. RMSProp technique [33] with a learning rate of 1e-3 is used to update the parameters of the BiLSTM-CRF model. While decoding, we predict the tag sequence **y**^∗^ that obtains the maximum score given by:

11 $$ {\mathbf{y}}^{\ast }=\underset{{\mathbf{y}}^{\prime }}{\mathrm{argmax}} score\left(\mathbf{X},{\mathbf{y}}^{\prime}\right). $$

The Viterbi algorithm [34] is used to infer the optimal tag path for efficiency considerations.

To further improve the PNER performance, the same post-processing rules as Luo et al. [20] and Campos et al. [35] are applied to pick the most likely entity mention back and correct incomplete entity mentions.

### Entity normalization

In this section, we explain how to map each recognized protein/gene mention to the corresponding ID in the UniProt or NCBI Gene KBs. Table 8 shows the pseudocode of our PNEN algorithm. It consists of the following two modules, (1) candidate ID generation and (2) entity disambiguation (i.e., pick the proper entity ID from all candidates as the mapping ID for each entity mention).

**Table 8:**
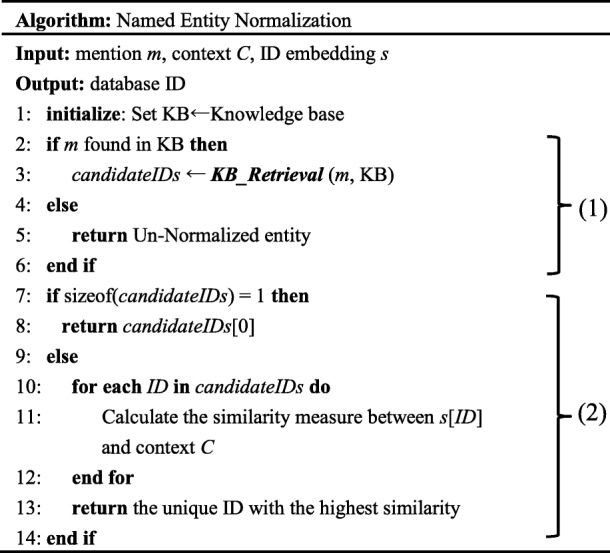
Pseudocode for PNEN Algorithm

Since pre-trained ID embeddings are required for entity disambiguation, how they are learned through structural knowledge of entities will be introduced first.

#### ID representation learning

KBs contain rich structural knowledge of entities (e.g., **name variations** and **entity ambiguity** as shown in Fig. 1), which can be formalized as constraints on embeddings and allows us to extend word embeddings to embeddings of entity ID. To this end, we adopt an autoencoder to learn the embedding of entity IDs based on Mention-Variant-ID structures provided by KBs.

The basic premises of the autoencoder are as follows: (i) entity IDs are sums of their variants; and (ii) entity mentions are sums of their variants. Take Fig. 1 as an example to illustrate. For the premise (i), entity ID “NCBI Gene: 7422” can be represented by the sum of its variants “VEGF (human)”, “MVCD1” and “VPF”. For the premise (ii), mention “VEGF” can be represented by the sum of its variants “VEGF (human)” and “VEGF (pig)”.

We denote mention embedding as *m*^(*i*)^ ∈ *ℝ*^*d*^, variant embedding as *v*^(*i*, *j*)^ ∈ *ℝ*^*d*^ and entity ID embedding as *s*^(*j*)^ ∈ *ℝ*^*d*^. *v*^(*i*, *j*)^ is that variant of mention *m*^(*i*)^ that is a member of entity ID *s*^(*j*)^. Mention embedding *m*^(*i*)^ is initialized by the average of the word embedding of its constituent words. We can then formalize our premises that the two constraints (i) and (ii) hold as follows:

12 $$ {s}^{(j)}=\sum \limits_i{v}^{\left(i,j\right)} $$

13 $$ {m}^{(i)}=\sum \limits_j{v}^{\left(i,j\right)} $$

The autoencoder consists of two parts, encoding and decoding. When encoding, it takes mention embeddings as input and unravels them to the vectors of their variants. And then, IDs can be embedded by the sum of their constituent variants. To unravel mentions to corresponding variants for ID representation learning, we introduce a diagonal matrix *E*^(*i*, *j*)^ ∈ *ℝ*^*d* × *d*^ to allow the mention *m*^(*i*)^ to distribute its embedding activations to its variants on each dimension separately. *E*^(*i*, *j*)^ satisfies the following condition ∑_*j*_*E*^(*i*, *j*)^ = *I*_*n*_ with *I*_*n*_ being an identity matrix. Therefore, the encoding process can be written as follow:

14 $$ {s}^{(j)}=\sum \limits_i{v}^{\left(i,j\right)}=\sum \limits_i{E}^{\left(i,j\right)}{m}^{(i)} $$

When decoding, it translates entity IDs back to mentions as follow:

15 $$ {\overline{m}}^{(i)}=\sum \limits_j{\overline{v}}^{\left(i,j\right)}=\sum \limits_j{D}^{\left(j,i\right)}{s}^{(j)} $$

where *D*^(*i*, *j*)^ ∈ *ℝ*^*d* × *d*^ is in analogy to the diagonal matrix *E*^(*i*, *j*)^ and used to distribute entity ID into its variants. ${\overline{m}}^{(i)}$ and ${\overline{v}}^{\left(i,j\right)}$ represent the entity mention and variant generated in the decoding process.

We align the decoded mention embedding with the original mention embedding to train the autoencoder. In addition, variant embeddings *v*^(*i*, *j*)^ and ${\overline{v}}^{\left(i,j\right)}$ obtained in both encoding and decoding parts are also aligned to strengthen the constraint on embeddings. Finally, our training objective for the autoencoder is to minimize the following equation:

16 $$ {\displaystyle \begin{array}{l} Loss=\alpha \cdot \left\Vert \sum \limits_j\left({D}^{\left(j,i\right)}\sum \limits_i{E}^{\left(i,j\right)}{m}^{(i)}\right)-{m}^{(i)}\right\Vert \\ {}\kern5.5em +\beta \cdot \left\Vert {E}^{\left(i,j\right)}{m}^{(i)}-{D}^{\left(j,i\right)}{s}^{(j)}\right\Vert \kern2.25em \forall i,j\end{array}}. $$

where *α* and *β* are weights and satisfy *α* + *β* = 1. We make *α* = *β* = 0.5 experimentally determined. With the help of this autoencoder, we thus encode structural knowledge of entities from UniProt and NCBI Gene KBs into ID embeddings, which are used as the input of the disambiguation model.

#### Candidate ID generation

In this module, for each entity mention *m* ∈ *M*, we aim to retrieve a candidate ID set *S*_*m*_ which contains possible IDs that entity mention *m* may refer to. To this end, we propose the ***KB Retrieval*** method for candidate ID generation, as shown in part (1) of Table 8.

***KB Retrieval*** method treats candidate ID generation as an information retrieval process, which takes mentions as queries and returns related IDs by the API resources provided by biomedical KBs. Here, UniProt official API [18] and NCBI-gene official API [19] are used to search for protein and gene IDs, respectively. To optimize for memory and run time, we keep top five results returned by ***KB Retrieval*** as candidate IDs for the entity mention.

#### Entity disambiguation

**Fig. 6:**
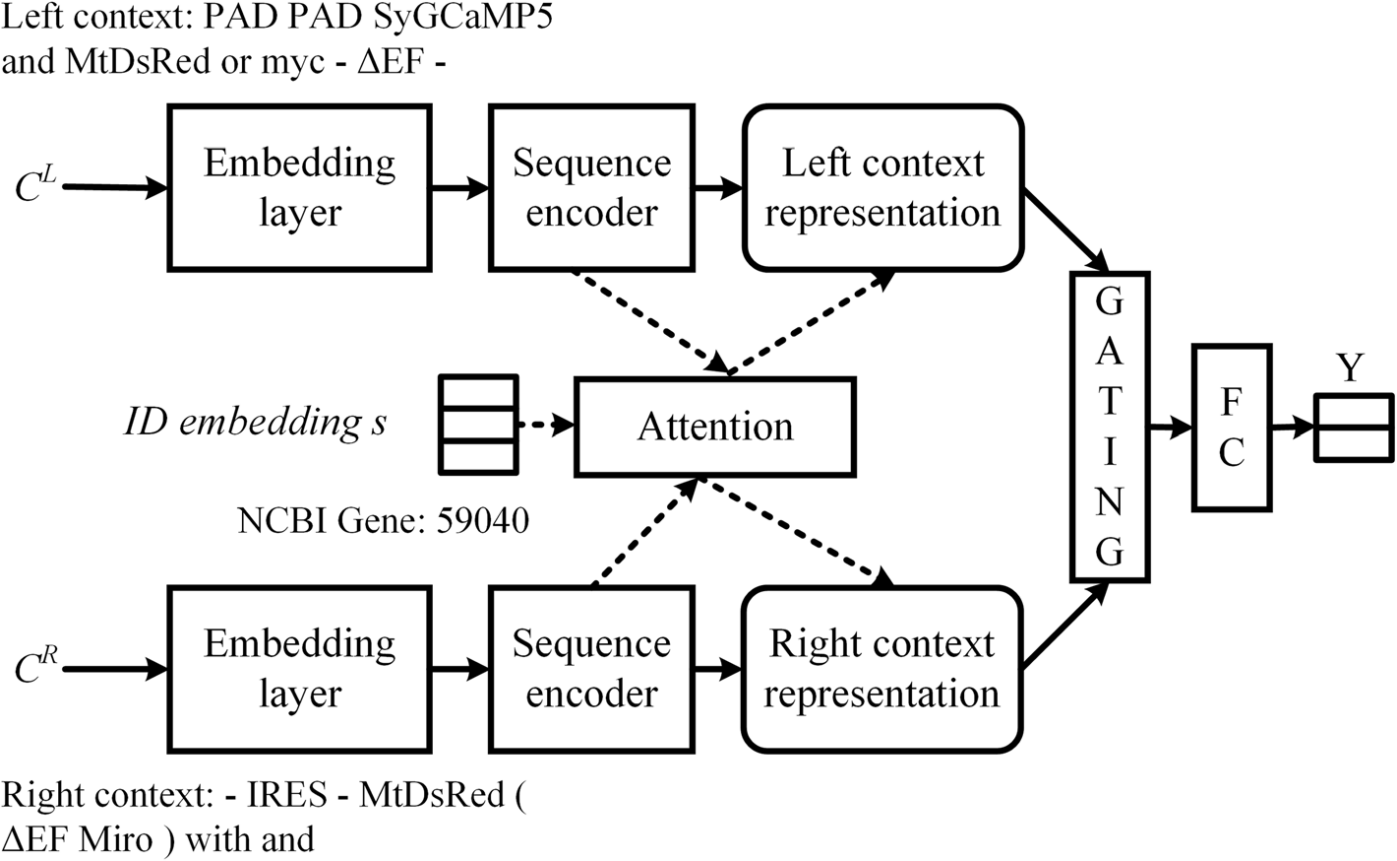
The architecture of our proposed disambiguation model. “ *C*^*L*^ ” represents the left context, “ *C*^*R*^ ” represents the right context, and “FC” represents the fully connected layer. The target entity is “**Miro1**” in Fig. 5. This figure takes candidate ID “NCBI Gene: 59040” as an example

Part (2) of Table 8 shows the process of entity disambiguation. In most cases, the size of the candidate ID set *S*_*m*_ of a mention is larger than one. We propose a disambiguation model, which is shown in Fig. 6, to rank the candidate IDs in *S*_*m*_ and pick the most likely one as the assignment result for the mention *m*.

Specifically, the model takes the left and right contexts where the entity mention appears and a candidate ID as inputs, and outputs a probability-like score for the candidate ID being correct. We set *n*_2_ as the length of the context sequence. The left context ${C}^L\in {\mathbb{R}}^{d\times {n}_2}$ and the right context ${C}^R\in {\mathbb{R}}^{d\times {n}_2}$ around the entity mention are first fed into a duo of sequence encoder (SE) for context representation learning. Take the left context as an example, the resulting hidden state of the *t*-th time step produced by the SE can be written as ${h}_t^{se}= SE\left({C}^L\right)$, where different architectures of SE will be compared in the experimental part.

For the semantic meaning of a sequence, the importance of each context word with respect to the candidate ID embedding *s* should be different. To this end, we fit the sequence encoder with a **knowledge-based attention** mechanism to focus on appropriate subparts of the input context. Following Eshel et al. [13], we use the pre-trained ID embedding *s* as the controller to calculate the normalized weight *α*_*t*_ ∈ [0, 1] for each hidden state ${h}_t^{se}$, which is then used to encode the entire sequence into a context representation *o*^*L*^ ∈ *ℝ*^*d* × 1^ as follow:

17 $$ {o}^L=\sum \limits_t{\alpha}_t{h}_t^{se}. $$

For each hidden state ${h}_t^{se}$, we use a feed forward neural network to compute its semantic relatedness with the candidate ID embedding *s*. The score function is calculated as follow:

18 $$ {e}_t=\tanh \left({W}_a^L{h}_t^{se}+{V}_a^Ls+{b}_a^L\right). $$

where ${W}_a^L\in {\mathbb{R}}^{1\times d},{V}_a^L\in {\mathbb{R}}^{1\times d}$ and ${b}_a^L\in {\mathbb{R}}^{1\times 1}$ are attention parameters to be learned during training.

After that, the attention weight *α*_*t*_ ∈ *ℝ*^1 × 1^ of each hidden state can be defined as follows.

19 $$ {\alpha}_t=\frac{\exp \left({e}_t\right)}{\sum_{k=1}^{T_k}\exp \left({e}_k\right)}. $$

In general, the contribution of left and right contexts should be different for the selection of different candidate IDs. For the purpose of dynamically controlling the flow of left context representation *o*^*L*^ and right context representation *o*^*R*^, a gating mechanism is also adopted as shown below:

20 $$ z=g\odot {o}^L+\left(1-g\right)\odot {o}^R. $$

21 $$ g=\sigma \left({W}_g{o}^L+{V}_g{o}^R+{b}_g\right). $$

where ⊙ denotes element-wise product between two vectors, *σ* is a sigmoid activation, and *W*_*g*_ ∈ *ℝ*^1 × *d*^, *V*_*g*_ ∈ *ℝ*^1 × *d*^, *b*_*g*_ ∈ *ℝ*^1 × 1^ are the model parameters that need to be trained.

Finally, we further concatenate the output of gating mechanism *z* ∈ *ℝ*^*d* × 1^ and the pre-trained ID embedding *s* as the final feature representation [*z*; *s*] and feed it to a classifier. The classifier consists of a two-layer fully-connected neural network (FC) with ReLU activation and an output layer with two output units in a softmax. The cross-entropy loss function is used as the training objective. Adagrad technique [36] with a learning rate of 5e-4 is applied to update parameters with respect to the loss function.

Note that, the disambiguation model picks the candidate ID which gets the highest score during the testing as the mapping ID for each entity mention *m*. However, in practice, some entity mentions do not have a corresponding ID in the knowledge base. Therefore, we also consider the assignment of a generic ID to them.

## Data Availability

The codes used in experiment are available at https://github.com/ningshixian/keras_bc6_track1. The API sources provided by biomedical KBs are publicly available from UniProt (https://github.com/cokelaer/bioservices) and NCBI Gene (https://github.com/biopython/biopython), respectively. GENIA Tagger is a preprocessing tool designed for biomedical text and available from the website (https://github.com/saffsd/geniatagger/).

## Abbreviations

biLMs: Bi-directional language models
BiLSTM-CRF: Bidirectional long short-term memory with conditional random field
BioNEN: Biomedical named entity normalization
BioNER: Biomedical named entity recognition
CNN: Convolutional neural network
ELMo: Embeddings from language models
*F*1: *F*1-score
FC: Fully-connected neural network
FNs: False negatives
FPs: False positives
GRU: Gated recurrent units
ID: Identifier
KBs: Knowledge bases
KFs: Knowledge features
LSTM: Long short-term memory
ML: Machine learning
*P*: Precision
PNEN: Protein/gene named entity normalization
PNER: Protein/gene named entity recognition
POS: Part-of-speech
*R*: Recall
SE: Sequence encoder
TPs: True positives

## References

[1] Yadav V, Bethard S. A survey on recent advances in named entity recognition from deep learning models. In: Proceedings of the 27th International Conference on Computational Linguistics. Santa Fe: COLING; 2018. p. 2145–58.

[2] Szklarczyk D, Morris JH, Cook H (2017). The STRING database in 2017: quality-controlled protein–protein association networks, made broadly accessible. Nucleic Acids Res.

[3] Arighi C, Hirschman L, Lemberger T, et al. Bio-ID track overview. In: Proceedings of BioCreative VI Workshop. Bethesda: BioCreative; 2017. p. 28–31.

[4] Leaman R, Lu Z (2016). TaggerOne: joint named entity recognition and normalization with semi-Markov models. Bioinformatics..

[5] Lu Y, Ji D, Yao X, Wei X, Liang X (2015). CHEMDNER system with mixed conditional random fields and multi-scale word clustering. J Cheminform.

[6] Ma X, Hovy E. End-to-end sequence labeling via bi-directional lstm-cnns-crf. In: Proceedings of the 54th Annual Meeting of the Association for Computational Linguistics. Berlin: ACL; 2016. p. 1064–74.

[7] Clark K, Luong MT, Manning CD (2018). Semi-Supervised Sequence Modeling with Cross-View Training. arXiv preprint arXiv:1809.08370.

[8] Lample G, Ballesteros M, Subramanian S, Kawakami K, Dyer C. Neural architectures for named entity recognition. In: Proceedings of the 2016 Conference of the north American chapter of the Association for Computational Linguistics: human language technologies. San Diego: NAACL; 2016. p. 260–70.

[9] Leaman R, Wei CH, Lu Z (2015). tmChem: a high performance approach for chemical named entity recognition and normalization. J Cheminformatics.

[10] Wei Chih-Hsuan, Kao Hung-Yu, Lu Zhiyong (2015). GNormPlus: An Integrative Approach for Tagging Genes, Gene Families, and Protein Domains. BioMed Research International.

[11] Tang B, Wu Y, Jiang M, Denny JC, Xu H. Recognizing and Encoding Discorder Concepts in Clinical Text using Machine Learning and Vector Space Model. CLEF (Working Notes). 2013;1179. http://ceur-ws.org/Vol-1179/.

[12] Zhang Y, Wang J, Tang B, Wu Y, Jiang M, Chen Y, Xu H. UTH_CCB: a report for semeval 2014–task 7 analysis of clinical text. In: Proceedings of the 8th International Workshop on Semantic Evaluation. Dublin: SemEval; 2014. p. 802–6.

[13] Eshel Y, Cohen N, Radinsky K, Markovitch S, Yamada I, Levy O. Named Entity Disambiguation for Noisy Text. In: Proceedings of the 21st Conference on Computational Natural Language Learning. Vancouver: CoNLL; 2017. p. 173–83.

[14] Ganea OE, Hofmann T (2017). Deep joint entity disambiguation with local neural attention. arXiv preprint arXiv:1704.04920.

[15] Li H, Chen Q, Tang B, Wang X, Xu H, Wang B, Huang D (2017). CNN-based ranking for biomedical entity normalization. BMC Bioinformatics.

[16] Shen W, Wang J, Han J (2015). Entity linking with a knowledge base: issues, techniques, and solutions. IEEE Trans Knowl Data Eng.

[17] Leaman R, Khare R, Lu Z (2015). Challenges in clinical natural language processing for automated disorder normalization. J Biomed Inform.

[18] Apweiler R, Bairoch A, Wu CH (2004). UniProt: the universal protein knowledgebase. Nucleic Acids Res.

[19] Edgar R, Domrachev M, Lash AE (2002). Gene expression omnibus: NCBI gene expression and hybridization array data repository. Nucleic Acids Res.

[20] Luo L, Yang Z, Yang P, Zhang Y, Wang L, Lin H, Wang J (2017). An attention-based BiLSTM-CRF approach to document-level chemical named entity recognition. Bioinformatics.

[21] Akhondi SA, Hettne KM, Van Der Horst E, Van Mulligen EM, Kors JA (2015). Recognition of chemical entities: combining dictionary-based and grammar-based approaches. J Cheminform.

[22] Peters ME, Neumann M, Iyyer M (2018). Deep contextualized word representations. arXiv preprint arXiv:1802.05365.

[23] Akbik A, Blythe D, Vollgraf R. Contextual string embeddings for sequence labeling. In: Proceedings of the 27th International Conference on Computational Linguistics. Santa Fe: COLING; 2018. p. 1638–49.

[24] Liechti R, George N, El-Gebali S, Götz L, Crespo I, Xenarios I, Lemberger T (2017). SourceData: a semantic platform for curating and searching figures. Nat Methods.

[25] Moen S, Ananiadou TSS. Distributional semantics resources for biomedical text processing. In: Proceedings of the 5th International Symposium on Languages in Biology and Medicine. Tokyo: LBM; 2013. p. 39–43.

[26] Zhao H, Lu Z, Poupart P. Self-Adaptive Hierarchical Sentence Model. In: Proceedings of International Joint Conferences on Artificial Intelligence. Buenos Aires: IJCAI; 2015. p. 4069–76.

[27] Lin Z, Feng M, Santos CN (2017). A structured self-attentive sentence embedding. arXiv preprint arXiv:1703.03130.

[28] Sheng E, Miller S, Ambite JS, Natarajan P. A neural named entity recognition approach to biological entity identification. In: Proceedings of the BioCreative VI Workshop. Bethesda: BioCreative; 2017. p. 24–7.

[29] Kaewphan S, Mehryary F, Hakala K, et al. TurkuNLP entry for interactive Bio-ID assignment. In: Proceedings of the BioCreative VI Workshop. Bethesda: BioCreative; 2017. p. 32–5.

[30] Kaewphan S, Hakala K, Miekka N, Salakoski T, Ginter F (2018). Wide-scope biomedical named entity recognition and normalization with CRFs, fuzzy matching and character level modeling. Database.

[31] Tsai Richard Tzong-Han, Hsiao Yu-Cheng, Lai Po-Ting (2016). NERChem: adapting NERBio to chemical patents via full-token features and named entity feature with chemical sub-class composition. Database.

[32] Tsuruoka Y, Tateishi Y, Kim JD, Ohta T, McNaught J, Ananiadou S, Tsujii JI (2005). Developing a robust part-of-speech tagger for biomedical text. Panhellenic conference on informatics.

[33] Tieleman T, Hinton G (2012). Lecture 6.5-rmsprop: divide the gradient by a running average of its recent magnitude. COURSERA.

[34] Viterbi A (1967). Error bounds for convolutional codes and an asymptotically optimum decoding algorithm. IEEE Trans Inf Theory.

[35] Campos D, Matos S, Oliveira JL, Sakurai S (2012). Biomedical named entity recognition: a survey of machine-learning tools. Theory and Applications for Advanced Text Mining. InTech, Rijeka, Croatia.

[36] Duchi J, Hazan E, Singer Y (2011). Adaptive subgradient methods for online learning and stochastic optimization. J Mach Learn Res.

